# Less Partnering, Less Children, or Both? Analysis of the Drivers of First Birth Decline in Finland Since 2010

**DOI:** 10.1007/s10680-022-09605-8

**Published:** 2022-02-14

**Authors:** Julia Hellstrand, Jessica Nisén, Mikko Myrskylä

**Affiliations:** 1 Centre for Social Data Science and Population Research Unit, University of Helsinki, Helsinki, Finland; 2 Max Planck Institute for Demographic Research, Rostock, Germany; 3 INVEST Research Flagship, University of Turku, Turku, Finland

**Keywords:** First births, Union formation, Union dissolution, Finland, Incidence-based multistate model, Counterfactual approach

## Abstract

In the 2010s, fertility has declined in the Nordic countries, most strikingly in Finland, and first births drive the decline. It remains unclear whether this decline results from decreased fertility within unions, changing union dynamics, or both. Thus, we investigated changes in the union–first birth dynamics from 2000 through 2018 in Finland using full-coverage population register data and an incidence-based multistate model. To do so, we calculated the yearly age-specific transition probabilities across states of single, cohabitation, marriage, and first births among 15- to 45-year-old childless men and women. We found lower fertility rates in unions after 2010, increasing dissolution rates amongst cohabiting couples, and long-term declines in the transition to marriage. Counterfactual simulations showed that, for the decline in first births since 2010, fertility within unions matters more (three-quarters) than union dynamics (one-quarter): that is, lower fertility in cohabitating and married individuals explained 42% and 13% of the decline, respectively, and decreasing fertility rates among couples entering cohabitation explained a further 17%. Decreasing marriage (19%) and cohabitation rates (2–4%) as well as higher union dissolution rates (6%) explained a smaller share of the first birth decline. The decline in first births was somewhat sharper among the lower social strata, but across strata the decreasing first birth transitions in unions explained most of the decline. To conclude, while changing union dynamics provide a partial explanation, postponing or foregoing fertility within unions represents the primary reason for the fertility decline.

**Supplementary Information:**

The online version contains supplementary material available at 10.1007/s10680-022-09605-8.

## Introduction

**Fig. 1:**
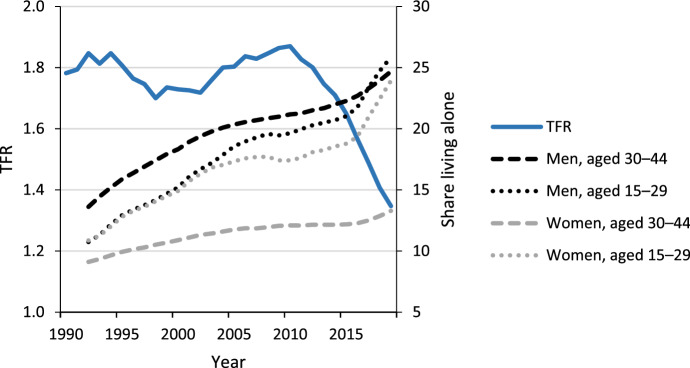
The total fertility rate (TFR) and the share living alone by age group and gender in Finland, 1990–2019. Source: Statistics Finland 2020, authors’ own calculations

In the 2010s, fertility sharply declined in many parts of Europe, particularly in the Nordic countries. The steepest of these declines occurred in Finland, where the total fertility rate (TFR) fell from 1.87 in 2010 to an all-time low of 1.35 in 2019 (Fig. 1; Official Statistics of Finland (OSF), 2019; Human Fertility Database, 2019). The Nordic fertility decline likely reflects declining lifetime fertility given that completed cohort fertility is projected to decline substantially for the first time in decades (Hellstrand et al., 2020, 2021). This projected decline is surprising, since the Nordic region previously featured a relatively high and stable cohort fertility, partly enabled by extensive social policy support provided by these countries intended to reconcile work and family life (Esping-Andersen, 2009; McDonald, 2000). Important findings identified first births as the main driver of the Nordic fertility decline (Hellstrand et al., 2020, 2021). In Finland, for instance, 75% of the decline is attributable to first births (Hellstrand et al., 2020, 2021). Therefore, a better understanding of why first births continue to decline should allow us to understand the general fertility decline.

Since most first births occur within unions (Jalovaara & Fasang, 2017; Kiernan, 1999), changes in union formation and union stability represent important factors in explaining fertility changes (Hiilamo, 2020). Finland and other Nordic countries serve as forerunners in shifting family formation patterns, such as lengthened single living during young adulthood, an increased progression to premarital cohabitation, and childbearing among cohabiting couples (Lesthaeghe, 2010; Surkyn & Lesthaeghe, 2004). The long-term increase in the share of the population living alone accelerated in more recent years in Finland. Specifically, in the 15- to 29-year-old age group, the share living alone rose from 21.0% in 2015 to 25.9% in 2019 among men and from 18.8 to 23.9% among women Fig. 1. Simultaneously, the number of childless cohabiting couples at older reproductive ages continues to increase. However, it remains unclear how the transitions into and out of unions and from unions to first births have changed over time, and whether these changes vary based on socioeconomic status.

The recent fertility decline occurred after the onset of the Great Recession, yet recession indicators insufficiently explain the fertility decline (Comolli et al., 2020). The fundamental reasons for the fertility decline remain unknown, but are hypothesised to be linked to broader uncertainty beyond the actual own circumstances (Vignoli et al., 2020a, 2020b) as well as to lifestyle factors (Rotkirch, 2020). Important questions as yet unaddressed include whether and how the decline in first birth rates since 2010 relates to changes in unions. That is, first births may be decreasing due to increasing difficulties related to forming or maintaining unions, due to decreasing tendency to transition to parenthood among couples, or due to a combination of these factors. By analysing whether the decline is driven by changes in unions versus changes in fertility within unions, we can also indirectly shed light on the pertinence of the reasons hypothesised to drive the decline. Investigating trends by socioeconomic status (SES) may be helpful here, as different SES groups may change their behaviour for different reasons. Previous research shows that first birth rates have declined across all female educational groups in the Nordic countries, albeit slightly faster among the least educated (Comolli et al., 2020). Yet, the role of unions was not taken into account in these analyses.

This study aims to examine the extent to which the decline in first birth rates in Finland results from changes in transitions between union states (single, cohabitating, and married) and changes in first birth rates within these states. We are particularly interested in changes in union formation (the transition from single to cohabitating and from cohabitating to married), union dissolution (the transition from cohabitating or married to single), and first birth rates within unions (the transition from cohabitating or married to first birth). Our research questions are as follows:

1. How have union–first birth dynamics changed over time?
2. Is the decline in first births driven by lower fertility in unions or by changes in union patterns?
3. How do these changes vary by socioeconomic status?

To answer these questions, we estimate the age-structured transition probabilities (single, cohabiting, married, and first births) among both men and women using full-coverage Finnish population register data, and work with the probabilities within the Markov chain multistate framework. We use an incidence-based multistate model and a counterfactual approach to estimate the impact of changes in unions and first birth transitions on declining first births in Finland from 2000 through 2018. By investigating union–first birth patterns for men and women at childbearing age, our study contributes to understanding the recent fertility decline in Finland. Because Finland is often viewed as a demographic forerunner (Andersson et al., 2009), trends here might provide insights into current fertility trends more broadly. Furthermore, the Finnish population registers are exceptional even within the Nordic context in that they include detailed, long-term information on nonmarital cohabitation.

## Background

### Union Dynamics and First Births: Theoretical Perspectives

Family demographic patterns have substantially changed in high-income countries in recent decades. Since the 1960s, fertility and marriage rates have decreased from high levels, divorce rates have increased from low levels, and nonmarital cohabitation and childbearing in cohabitation have become widespread (Lesthaeghe, 2010). Furthermore, childlessness is becoming more important in shaping fertility developments in high-income countries (Kreyenfeld & Konietzka, 2017; Miettinen et al., 2015), although much of the variation across countries in total fertility currently still depends on variation in second and higher-order births (Frejka, 2008; Zeman et al., 2018). In addition to the increased availability of efficient contraception fuelling early fertility declines beginning in the 1960s (Goldin, 2006), these changes have often been attributed to changes in gender roles and shifts in attitudes and norms. More recently, broader economic uncertainty was also put forth as playing a role in fertility and marriage declines.

The shift from a negative to a positive relationship between female employment and fertility in the late twentieth century (Ahn & Mira, 2002; Brewster & Rindfuss, 2000) gave rise to theories predicting a U-shaped trend in fertility levels over time (Myrskylä et al., 2009, 2011; Esping-Andersen & Billari, 2015). According to these theories, improvements in gender equality would cause fertility to recover and would also strengthen the family in terms of increasing union formation and decreasing union dissolution (Goldscheider et al., 2015). As long as traditional gender roles prevail within families, the increase in women’s labour force participation is expected to increase work–family conflict among women, thereby potentially depressing fertility, increasing the risk of union dissolution, and even discouraging union formation. By contrast, when men’s involvement in the family increases, resulting in less unequal sharing of domestic chores, women’s work–family conflicts are expected to decrease, with potentially strengthening effects on families, including increased fertility (Anderson & Kohler, 2015).

This U-shaped prediction linking gender equality and fertility, however, is largely fuelled by cross-sectional evidence, and longitudinal analyses do not provide strong support for the prediction (Kolk, 2019). Although the gender revolution may have helped to prevent cohort fertility from decreasing to very low levels, there is little evidence that it would have increased cohort fertility (Frejka et al., 2018). One reason for the lack of a strong positive association may be that increasing gender equality could change men’s incentives for having children in ways that are poorly understood, and some of these forces may be negative. At present, cohort fertility in the Nordic countries is predicted to decline for the first time in decades, further challenging these predictions of a positive relationship (Hellstrand et al., 2020, 2021). Since there have been no signs of weakening gender equality in families or significant changes in family policies in the recent decade, the Nordic fertility decline calls for alternative explanations.

The second demographic transition (SDT) theory represents another central theory to explain family changes in recent decades. While theories linking gender equality and fertility predict a reversal towards ‘more family’, SDT predicts sustained low fertility and a continuously weakening role of the family. According to SDT, changes to family formation patterns associate with shifts in attitudes and norms towards greater individual autonomy and self-actualisation (Surkyn & Lesthaeghe, 2004). The central idea is that the departure from institutional control and authority paves the way for greater individual autonomy in decision-making, whereby the emergence of ‘higher-order needs’ drives fertility decisions (Mills et al., 2011). Hence, the importance of marriage decreases, alongside the rearing of a child increasingly becoming a conscious choice taken to achieve greater personal self-fulfilment (Van De Kaa, 1987). Childbearing can be viewed as a competing event against other life goals, with women more likely postponing childbearing if they associate children with impeding their individual autonomy (Liefbroer, 2005). Changes in values are considered to result in both increased childlessness and decreases in higher parity births, given that the latter can result in further obstacles for self-fulfilment (Lesthaeghe, 2014). Additionally, greater emphasis is placed on the quality of relationships, consequently leading to the postponement of commitments and increasing tendency for unions to dissolve. According to SDT theory, shifts in demographic behaviours are first adopted by the more highly educated who possess more advanced post-materialist values, which then spread to the rest of society (Lesthaeghe & Surkyn, 1988).

Finally, economic constraints and economic uncertainty serve as important factors explaining trends in family formation. According to microeconomic theories, couples with greater socioeconomic resources at their disposal are more likely to have (more) children given the direct costs associated with children, when such costs are not counterbalanced by higher opportunity costs (Becker, 1993). In advanced societies, individuals tend to postpone childbearing during economically uncertain times and favour it during times of economic growth (Sobotka et al., 2011). Of all parities especially first births have been affected negatively by rising economic uncertainties (Blossfeld & Hofmeister, 2006). Economic uncertainty may also have negative effects on union formation and stability (Mills & Blossfeld, 2003).

However, the fact that the fertility decline continued in the Nordic countries during the 2010s despite macro-economic recovery has been argued to highlight the need for a broader framework of perceived uncertainty to explain fertility changes (Comolli et al., 2020). Some hypothesise that uncertainty in people’s lives stemming from globalisation dynamics, new technologies, and media channels since the onset of the Great Recession may have increased in the 2010s (Vignoli et al., 2020a, 2020b). In the narrative framework, expectations and imaginings about the future, which arise from individuals’ past experiences and shared narratives (from peers, social media, or others) and extend beyond actual economic and labour market indicators, may importantly influence fertility decision-making (Vignoli et al., 2020a, 2020b) and marriage intensions, while forming a cohabitation appears to be more compatible with such uncertainties (Guetto et al., 2020; Mills & Blossfeld, 2013). The reason why cohabitation is expected to differ from marriage and childbearing in this respect is that a cohabitation requires much less commitment than a child or a marriage, as a cohabitation can be fairly easily ended.

In this study, we compare the trends in union patterns and first births in Finland within a conceptual framework built on these different lines of theory. Although this study does not aim at providing direct evidence to test these theories, it may provide some insights that are also theoretically interesting: some explanations of the fertility decline may more strongly operate through suppressing union formation or marriage, or inciting union dissolution than suppressing fertility in unions, and different SES trends may give some further hints on the mechanisms behind the fertility decline. Based on the SDT narrative, we would expect to observe a decrease in childbearing within unions and/or changing partnership dynamics (delayed unions, increasing cohabitation with a lower probability of a first birth compared to marriage, and increasing union dissolution), which are more strongly prevalent among the more highly educated. This stems from the assumption that new demographic behaviours are adopted first by the more highly educated (Lesthaeghe & Surkyn, 1988). However, although many of the changes predicted by SDT have been observed over the past five decades, currently childlessness, never partnering, union dissolution, and childbearing in cohabitation are all more strongly prevalent in lower SES groups (Jalovaara & Fasang, 2017; Jalovaara et al., 2019; Perelli-Harris et al., 2010). It can thus be debated that how relevant the SDT framework is for explaining the current fertility patterns.

A decline in first births primarily explained by decreasing fertility and marriage intensities—rather than by shifting cohabitation patterns—would be in line with the uncertainty theory, as cohabitation may not be affected by uncertainty to the same extent as marriage and the entry into parenthood. There is little consensus on how perceived uncertainty can be expected to affect union dissolution (Bastianelli & Vignoli, 2021). Actual economic constraints may increase the risk of separation due to economic stress, and similarly, perceived uncertainty could also bring forth stress that strains the relationship. The reluctance to progress to a more binding relationship (i.e. marriage or parenthood) due to uncertainty could be reflected in the dissolution of cohabitation especially if the partners’ disagree on the issue. On the other hand, marriage brings predictability to the future, and hence, perceived uncertainty may not affect divorce risks. Changes in family formation patterns more strongly driven by lower SES groups may reflect obstacles to family formation due to economic constraints or uncertainty. As lower SES groups face more difficulties in finding a partner (Jalovaara & Fasang, 2017), their union formation and consequently first births may be delayed as compared to the high SES groups. They may also have a lower first birth rate because they are more likely to experience union dissolution once they have entered a union (Jalovaara, 2013). However, economic uncertainty may arise in spite of the actual own economic situation (Vignoli et al., 2020a), and consequently, a homogenous change among the SES groups is not necessarily evidence against uncertainty as an explanation for the decline.

### Union Dynamics and First Births: Empirical Observations in Finland

Cohabitation currently represents a common first step in family formation in Finland. The vast majority of all first unions (over 90%) begin as cohabiting unions (Jalovaara, 2012), and Finland’s proportion of 20 + year-old people living in cohabiting unions is among the highest in Europe (Corselli-Nordblad & Gereoffy, 2015). Cohabiting couples exhibit much higher separation rates than married couples, but the separation rate decreases with cohabitation length, and most cohabiting couples which do not separate eventually marry (Jalovaara, 2013). When it comes to divorce, the crude divorce rate has slowly declined after reaching its peak of 2.9 in 1989 (Eurostat, 2021).

First births are becoming increasingly disconnected from marriage, as first births are increasingly more likely to occur within cohabitation (Kennedy & Bumpass, 2008; Thomson & Eriksson, 2013). The share of births to unmarried women in Finland increased from 33% in 1995 to 45% in 2018, consequently placing Finland currently above the average of 42% across 28 OECD countries, but clearly lower than in other Nordic countries (OECD, 2021b). By 2018, the mean age at first birth reached 29.4 years and the mean age at first marriage 31.7 years among Finnish women (Official Statistics of Finland (OSF), 2018a, 2018b). Although first births are increasingly born to cohabiting couples, marriage and childbearing are still interconnected so that cohabiting couples who have children often marry (Rotkirch & Miettinen, 2017). A study from Iceland concludes that the order of events accounts for this change: marriage now takes place after rather than before childbearing, although marriage does not seem to be declining (Jónsson, 2020). However, married couples still have stronger childbearing intentions (Miettinen & Rotkirch, 2008) and higher first birth rates than cohabiting couples (Jalovaara & Miettinen, 2013).

Compared to other high-income countries, Finland exhibits high rates of ultimate childlessness (Sobotka, 2017), and most Finns without children of their own have never lived in a stable, long-term cohabitating union (Jalovaara & Fasang, 2017; Saarela & Skirbekk, 2019). Hence, difficulties encountered in finding a partner represent one likely factor explaining high rates of childlessness in Finland. Furthermore, the contemporary gender imbalance in education, such that women are better educated than men, is particularly pronounced in Finland (OECD, 2021a). This new gender imbalance in education limits the opportunities of finding a suitable partner particularly among the least educated men who now largely outnumber women without a degree, since historically women have formed unions with men at least as well educated as themselves (Mäenpää & Jalovaara, 2015; Van Bavel, 2012). A stable partnership continues to be a strong prerequisite to childbearing also in Finland (Jalovaara & Fasang, 2017). Marriage serves as an indicator of greater union stability as opposed to cohabitation and proof of a commitment, and married men and women are much less likely to remain childless than cohabiting men and women (Saarela & Skirbekk, 2019).

There are pronounced differences in union histories and the transition to parenthood between educational groups in Finland. The lower educated tend to form unions and have their first birth at younger ages than the higher educated (Jalovaara & Fasang, 2020; Nisén et al., 2014). Socioeconomic resources of both genders promote cohabitation and particularly marriage, and lower the risk of union dissolution (Jalovaara, 2012, 2013). For instance, over one third of Finns with a tertiary education had married their first cohabiting partner and were still in that marriage at age 39, while the corresponding share was 13% for those with only compulsory education (Jalovaara & Fasang, 2017). Ultimate childlessness has recently sharply increased among the less educated, and consequently, the educational gradient in childlessness among women has reversed (Jalovaara et al., 2019). Currently, both the least educated men and women are most likely to remain childless: in a late 1960s Finnish cohort consisting of individuals who completed only a basic education, over 30% of women and over 40% of men remained childless at age 40–41 (Rotkirch & Miettinen, 2017). This negative relationship between educational attainment and ultimate childlessness appears strongly driven by lower chances of the less educated to form stable unions (Nisén et al., 2018; Saarela & Skirbekk, 2019). Further, the least educated are more likely to have children in cohabitating unions while the more highly educated tend to exhibit higher first birth rates within marriage (Jalovaara & Andersson, 2018). Still, little is known about how fertility declines in the 2010s are associated with changes in unions and whether these potential changes vary based on SES.

## Data and Methods

### Data

In this study, we used Finnish national longitudinal population register data compiled at Statistics Finland (permission no. TK-52-1119-17). The register data were linked to different register sources such as information on childbirths, housing and educational attainment through personal identification numbers, offering full-coverage of the entire Finnish population. The study population consists of all childless men and women aged 15–45-years-old permanently living in Finland on the last day of each year from 2000 through 2018. Individuals were followed until they had a first biological child or until they reached the age of 45. In total, the study population consists of 2 532 375 individuals and 23 847 070 person-years. Less than 0.06% of all first births were linked to two biological mothers/fathers. Consequently, the true parent for these children remained unknown. We excluded from our study 388 individuals linked to such a first birth.

For each individual, data include personal information on family status (single, cohabiting, or married) at the end of each calendar year. Statistics Finland defines cohabitation as a union of two unmarried adults of the opposite sex aged 18 or older who have been living in the same dwelling for at least three months, who are not siblings or differ in age by 16 or more years (Official Statistics of Finland (OSF), 2021). An individual is considered single if s/he is not living in a cohabiting or married union. Among the study population, 2.1% of men and 1.5% of women (446 787 observations) had missing information for family status (institutionalised population and/or otherwise unclassified) and were thus excluded from the study.

We formed yearly transitions for all individuals in the study population for whom personal information was available for two consecutive years. Information for two consecutive years was missing for all first entries into the study population (2 517 735 observations) and for individuals absent from the Finnish population during some period from 2000 through 2018 (130 467 observations). Furthermore, to avoid challenges related to incomplete educational data and an unknown number of unregistered first births to non-native Finns, we excluded individuals born abroad^1^ (1 280 473 observations for 229 670 individuals). In total, we identified 19 468 815 yearly transitions between states (single, cohabitating, married, and first birth) for 2 125 172 individuals beginning in 2000. Among these, 740 537 were transitions to first births^2^, and 2 911 543 were transitions between partnership states. Appendix Table 1 provides descriptive information about first births and partnership transitions in more detail.

We also estimated the transition probabilities based on SES. We considered four categories of educational attainment—primary, secondary, lower tertiary, and higher tertiary. Primary-level includes those who completed at most a lower secondary level of education (ISCED 0–2), while secondary level refers to those who completed upper secondary and post-secondary non-tertiary levels of education (ISCED 3–4). Lower tertiary includes short-cycle tertiary education and a Bachelor’s degree or the equivalent level (ISCED 5–6), while higher tertiary refers to those who completed a Master’s degree, doctoral degree or the equivalent level of education (ISCED 7–8). We used income as a complement to education as a robustness check to overcome the limitations related to using educational attainment as an explanatory variable in the period analysis—that is, currently, less educated groups include those who will later attain more advanced degrees. The income variable refers to each individual’s annual income subject to state taxation and includes both earnings and social-security benefits. Four income groups were formed based on income quartiles stratified by age, year, and gender. Because those enrolled in educational programmes in particular are known to exhibit lower birth risks (e.g. Kravdal, 1994), we performed a sensitivity analysis which excluded students (shown in the appendix).

### Methods

We used a Markov chain multistate approach, which describes the transitions between a given set of states using transition probabilities (Briggs & Sculpher, 1998). A Markov chain evolves in discrete time and moves step-by-step from state $i$ to state $j$, with the property of being memoryless. That is, the probability of each transition depends only on the state attained in the previous step and not on the history of events (Kemeny & Snell, 1971). The transition probabilities from state *i* to state *j* at a specific age and time are defined as

$$ p_{ij} \left( {age,t} \right) = pr\left( {State_{t} = j{|}State_{t - 1} = i; age_{t - 1} } \right). $$

**Fig. 2:**
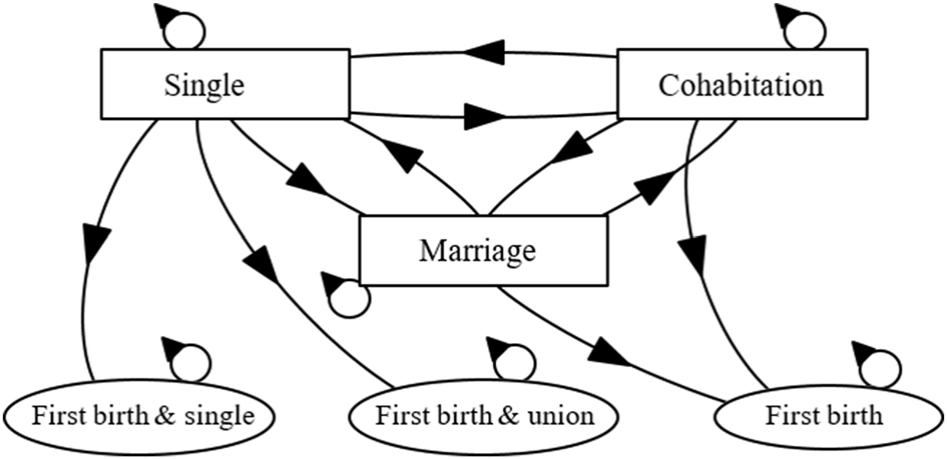
State transition diagram for the Markov chain

The step size in our analyses is one year.^3^ Our state space includes the states of ‘single’, ‘cohabitating’, ‘married’, and ‘first birth. An illustration of the state space and the transitions between these states appears in Fig. 2. In our analysis, we distinguish between the transitions from ‘single’ to ‘first birth and single’ and ‘first birth and union’ in order to distinguish single parents from couples who begin cohabitating closer to the first birth event. The first birth event represents an absorbing state, meaning that once entered it cannot be left. All other states are nonabsorbing (transient) states. We estimated the yearly age-specific transition probabilities for each of the given set of states between the ages of 15 and 45 from 2000 through 2018 as

$$  p_{{ij}} \left( {x,t} \right) = \frac{{\# {\text{individuals}} \;{\text{in}}\; {\text{state}}\;j\;{\text{in}} \;{\text{year}}\; t\; {\text{aged}}\;x\; {\text{and}}\; {\text{in}} \;{\text{state}}\;i\;{\text{in}}\;{\text{year}}\;t - 1}}{{\# {\text{individuals}}\;{\text{aged}}\;x\;{\text{in}}\;{\text{state}}\;i\;{\text{in}} \;{\text{year}}\;t - 1}} $$

using simple cross tabulations.^4^ The probabilities were estimated separately for men and women, as well as for educational and income groups, respectively.

We used the estimated transition probabilities and counterfactual simulation^5^ to calculate what proportion of the decline in first births was attributable to changes in union dynamics versus the decline in fertility within unions.^6^ (For specific details, see the Technical appendix.) First, we calculated the age-specific first birth rates that would have been observed if the population in 2010 would have experienced the 2010 transition rates in the period from 2010 through 2018. We labelled this scenario ‘constant probability births’. Using the age-specific first birth rates, we calculated the proportion ever having a first birth according to a life-table approach. Second, we calculated the age-specific first birth rate and the proportion ever having a first birth that we expect to have observed if the population in 2010 would have experienced the observed changes in transition rates in the period from 2010 through 2018. We labelled this scenario ‘natural course births’. We decompose the difference between these two scenarios by changing the transition probabilities one at a time. For education groups, we adjusted the procedure to take into account that the study population progresses to higher education levels over time. Additional details appear in the Technical appendix.

## Results

### Age-Specific Transition Probabilities Between States

**Fig. 3:**
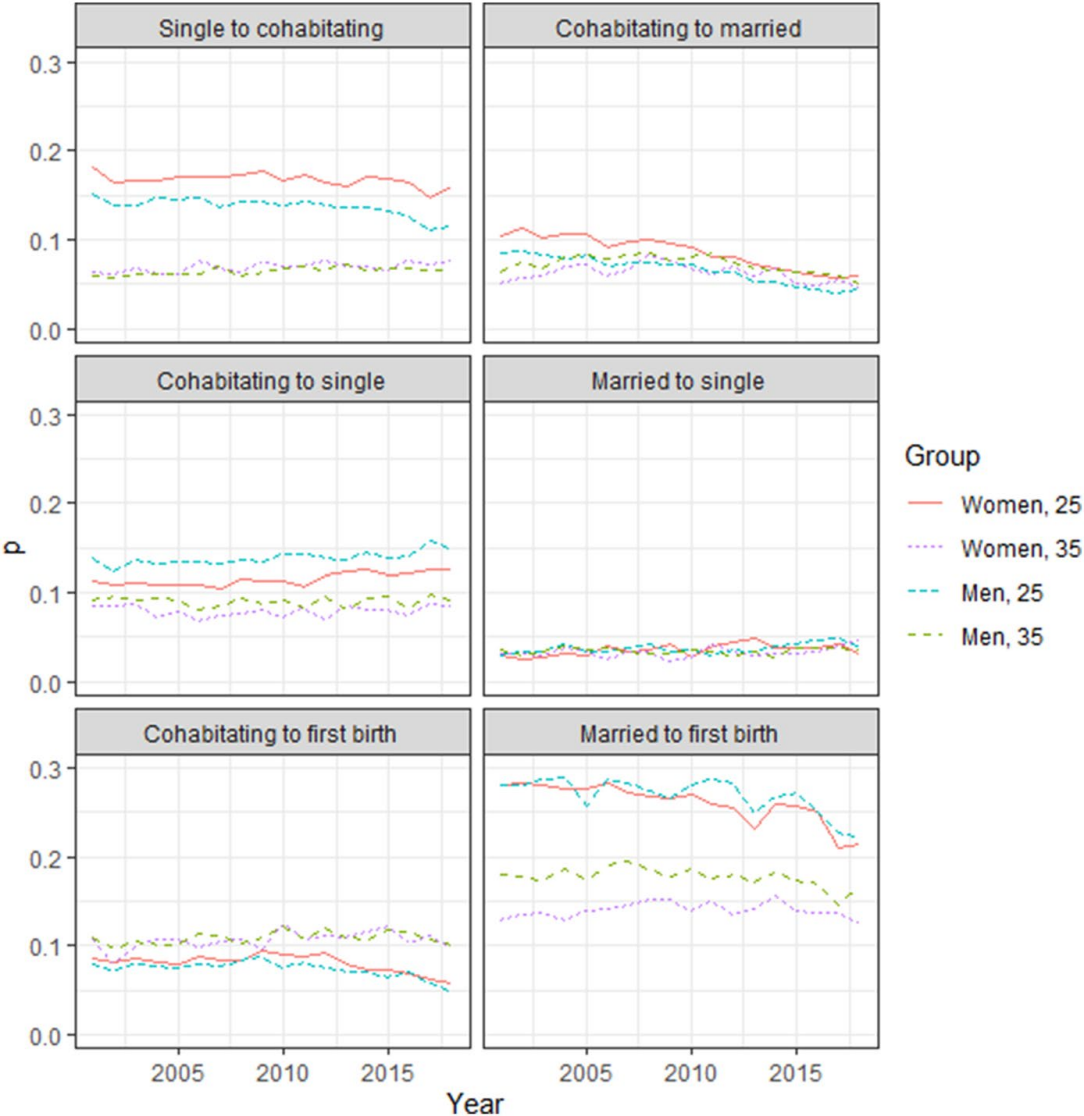
Transition probabilities for single, cohabitating, and married individuals, and the first birth for childless women and men aged 25 and 35 in Finland, 2001–2018

First, we explored how the age-specific transition probabilities have changed over time. The selected yearly age-specific transition probabilities between single, cohabitating, and married individuals, and first births for childless men and women in Finland from 2000 through 2018 appear in Fig. 3. We show the developments for two selected age groups, 25 and 35, which represent the patterns among younger and older age groups, respectively. The age-specific transition probabilities for all events appear in Appendix Fig. 7.

#### Union Formation

Since the early 2000s, the probability of forming unmarried, cohabitating unions has remained relatively stable across all ages. However, we observed a drop for the most recent years (2015–2018) among the younger ages, that is, at age 25 when the transition probability fell from 14 to 12% for men. Among women, a drop was observed only among those younger than 25. Thus, the yearly probability of remaining single has recently increased among younger individuals. The probability of marrying has exhibited a more long-term decline at nearly all ages. For instance, the yearly probability of marrying at age 25 among cohabiting women has, since the early 2000s, decreased from 11 to 6% in 2018, peaking in 2008 at 8% for age 35, and then falling to 5% in 2018. Additionally, the low probability of (directly) marrying among single individuals has decreased.

#### Union Dissolution

Married couples exhibited a lower rate of union dissolution compared to cohabiting individuals at all ages. The rate of union dissolution among married couples remains similar across all age groups, remaining relatively stable over the period of interest. Among cohabiting couples, the rate of union dissolution was higher in the younger age groups. In recent years, the probability of a union dissolving among cohabiting men and women increased slightly at younger ages. That is, at age 25, the transition probability increased from 11 to 13% among women and from 14 to 15% among men from 2010 to 2018. Furthermore, the transition from marriage to cohabitation remains rare.

#### Transition to First Births

The age pattern in the transition to first births differs widely for cohabiting and married couples. First birth rates within cohabitating couples were highest during the early 30 s when around 10% of men and women who were cohabiting in 2017 experienced their first births in 2018. First birth rates among married men and women where highest at very young ages. Thus, first birth rates were several times higher at younger ages among married versus cohabiting couples, a difference that persisted until the mid to late 30 s. Since 2010, first birth rates have decreased among both cohabiting and married couples for nearly all ages, but more distinctly at younger ages. The decrease was, however, less pronounced among married women. For instance, from 2010 through 2018, the first birth rate at age 25 decreased from 0.27 to 0.21 among married women and from 0.09 to 0.06 among cohabiting women. Furthermore, the low probability of transitioning from single to first birth—specifically the transition from single to first birth and entering a union—has decreased. We observed a sharp increase in the probability of remaining either as a cohabiting or a married couple without transitioning to a first birth.

### Transition Probabilities by Socioeconomic Status (SES) Groups

**Fig. 4:**
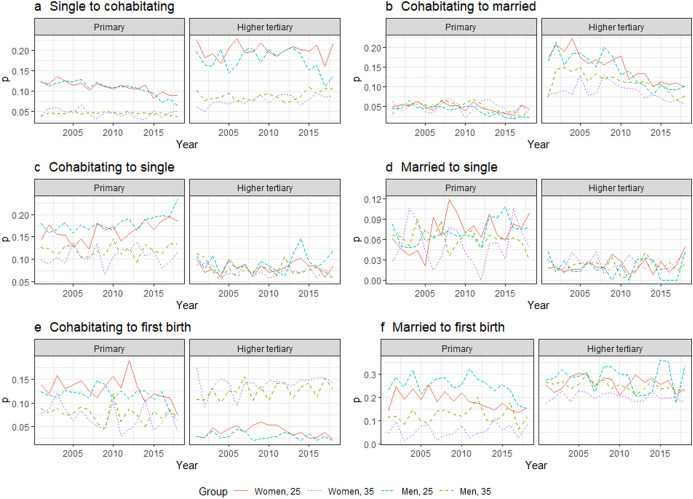
Transition probabilities for single, cohabitating, and married individuals, and for first births among childless women and men in 2001–2018 by level of education at ages 25 and 35

We further explored whether changes in partnering and first birth transitions were more pronounced in some SES groups than others (Fig. 4). We show the results for the lowest and highest SES groups.

The decrease in the transition to cohabitation observed at younger ages in the general population was visible primarily among less educated grosups of women and men. Over years 2010—2018, the probability of forming a cohabiting union at age 25 declined for primary educated men and women by 5 and 3 percentage points, respectively. This is a 45% (men) and a 27% (women) decline of the levels observed in year 2010. We observed no change in cohabitation rates among women with tertiary-level education, but found a decline (6 percentage point which is 30% of the level in 2010) for the relatively small group of higher tertiary-level educated 25-year-old men. Furthermore, a long-term decreasing trend in the transition from cohabitation to marriage was observed across all SES groups, but appeared somewhat stronger among the more highly educated. For instance, the transition probability from cohabitation to marriage at age 25 remained low at around 0.05 for primary educated women, but fell from 0.18 to 0.10 for higher tertiary educated women.

We also observed a slight increase in the transition from cohabitation to being single primarily among the least educated men and women. Moreover, we observed no change in the transition from marriage to being single in any of the educational groups, except for a small potential increase among the least educated at younger ages.

Finally, we find that first birth rates have decreased both among cohabiting and married men and women across all SES groups. However, the decrease was less pronounced in the small group of married women in the lowest educational group.

### Contributions to Declining First Births, 2010–2018

**Fig. 5:**
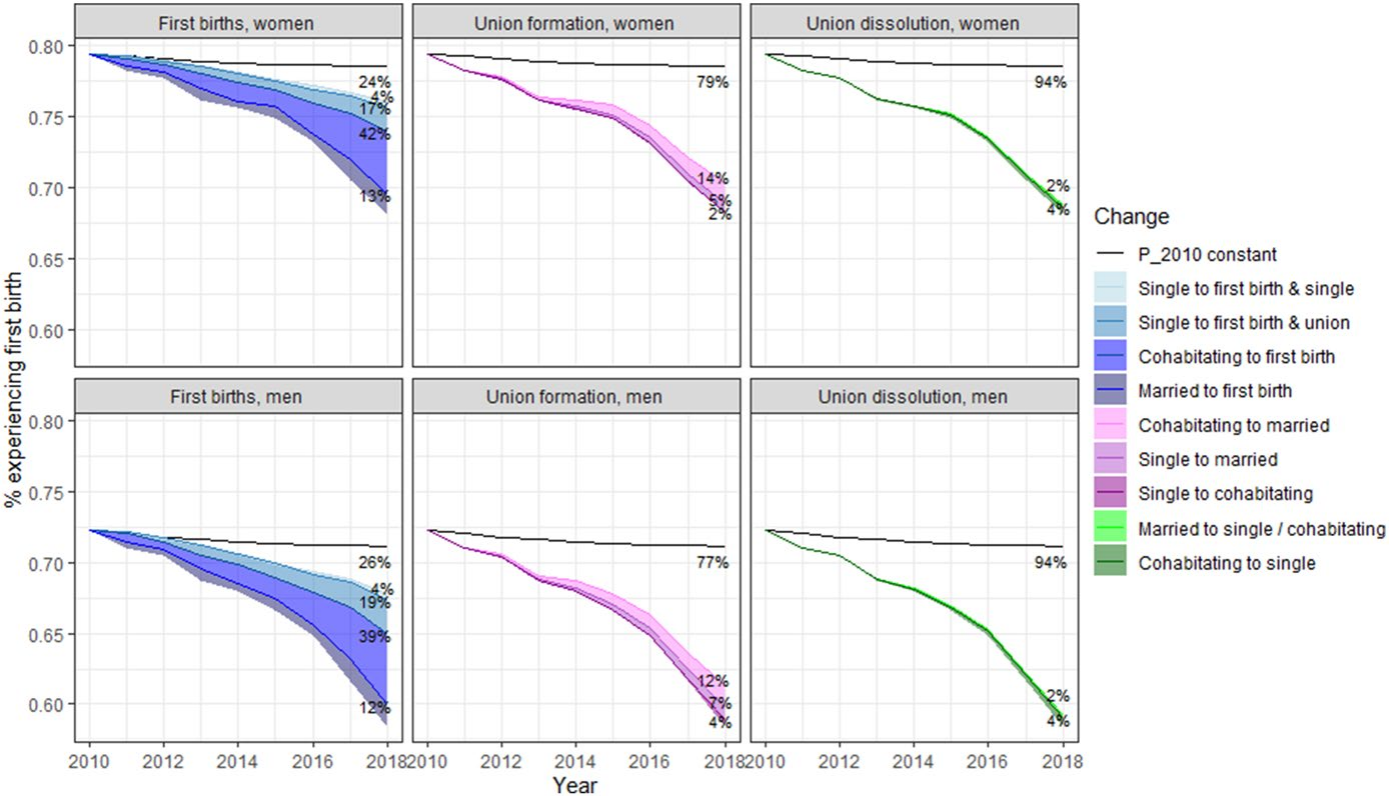
Contributions of declining first births, changes in union formations, and changes in union dissolutions to the decline in the percentage experiencing first births based on the first birth rates in 2010–2018. The black solid line indicates the percentage experiencing a first birth that would have been observed if the population in 2010 would have experienced the 2010 transition rates in the years 2010 through 2018. Shaded areas indicate how much the decline in first births would have been dampened if the corresponding transition probabilities would not have changed

Figure 5 shows the contributions of the changes in first birth transitions, union formations, and union dissolutions to the decline in the number of first births from 2010 through 2018. We use the percentage experiencing a first birth, a synthetic age-standardised measurement indicating the proportion that expected to experience a first birth based on the observed rates. The observed changes in the transition probabilities led to a decline in the share experiencing a first birth to 68.1% for women and to 58.4% for men. This natural course scenario matches well with the true observed first birth rates (Appendix Fig. 10). If the transition probabilities remained stable (i.e. as observed in 2010) across years, the share experiencing a first birth based on the age-specific first birth rates in 2018 would have reached 78.6% for women and 71.2% for men.

If first birth transitions (whether among single, cohabitating, or married individuals) had not decreased, 76% of the observed decline in the share experiencing a first birth among women would have been dampened. The largest contributions originated from cohabiting women (42%), followed by women who were single at the end of the year, but who experienced their first birth and lived in a union at the end of the following year (17%) and married women (13%). The decrease in single motherhood also insubstantially contributed (4%). Furthermore, if union formation had remained stable, the decrease would have been dampened by 21%. Decreasing marriage rates appeared vastly more important (19%) than decreasing cohabitating rates (2%). In addition, the small increase in dissolution rates contributed modestly (6%). It is also possible that the couple did not marry because they did not have the first birth in the first place. Among men, the results were largely similar, but, the decrease in cohabitation rates, for instance, was slightly more important among men than among women.

#### Contribution by Socioeconomic Status Groups

The results of the decomposition are affected by both the probabilities of transitioning between different states, as well as the population composition, which both differ between SES groups and contribute partially to findings between groups. In order to more intuitively grasp the compositional effects of transitions between states in the counterfactual simulation, we show the distribution of union states in 2009 and 2017 for the lowest and highest educational groups in (Appendix Fig. 8). Notably, the proportion single is much higher among the lower educated, and more people are cohabitating and married among the higher educated, throughout the study period. When it comes to changes in the last decade, we observe that among the lower educated, the proportion single has increased while the proportion in unions (both cohabiting and married) has decreased. Among the higher educated, the proportion single has remained relatively stable, but the proportion married decreased and the proportion cohabiting increased.

**Fig. 6:**
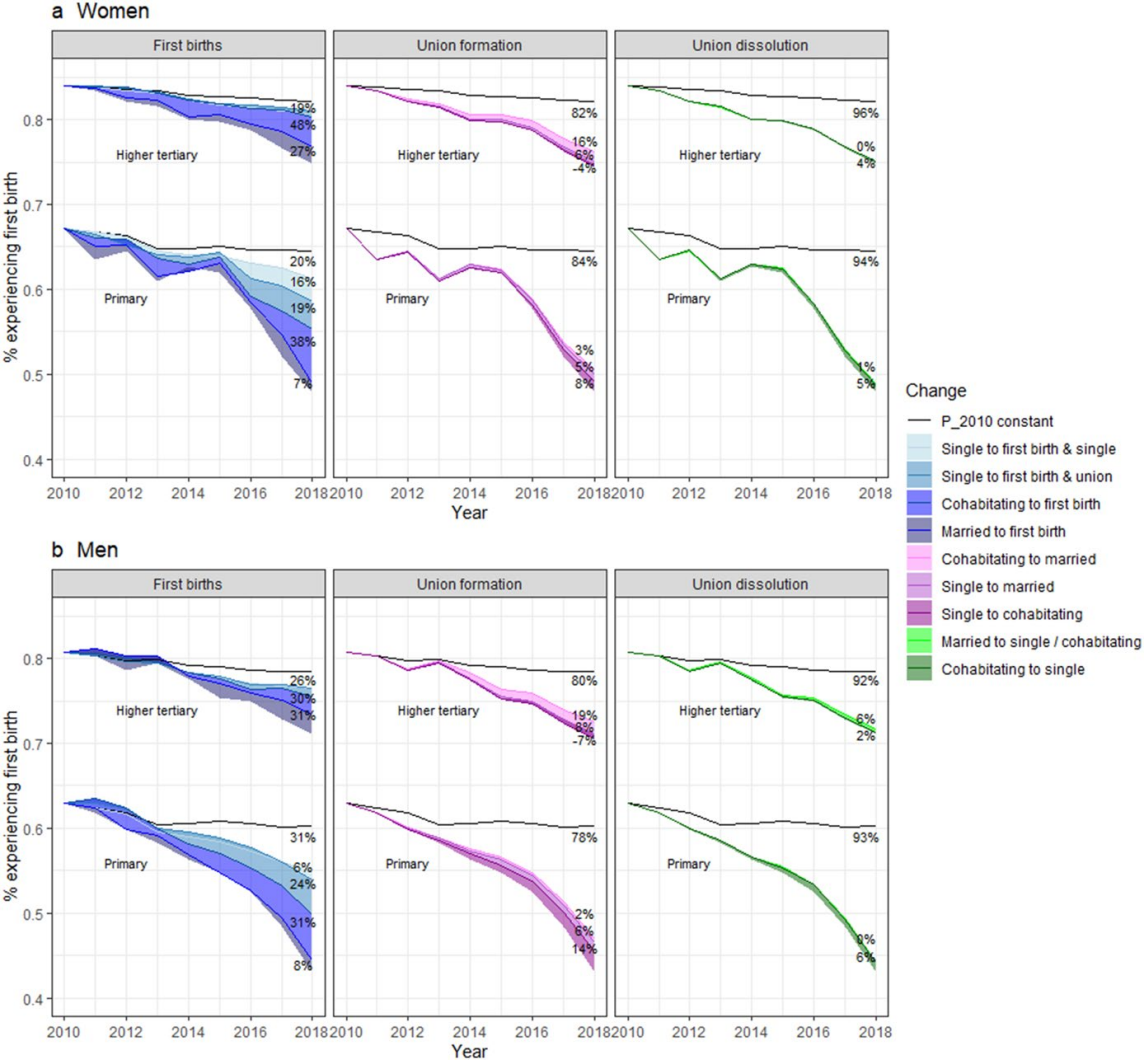
Contributions of declining first births, changes in union formations, and changes in union dissolutions to the decline in th percentage experiencing first births based on the first birth rates in 2010–2018 by education groups. The top curve shows the results for the higher tertiary education groups, while the bottom curve shows the results for the primary-level education groups

Figure 6 shows the contributions to the first birth decline based on education. The total decline was larger in the lower SES groups. Specifically, the share ever experiencing a first birth fell from 2010 through 2018 from 65 to 48% for women with primary education and from 82 to 75% for women with higher tertiary education. In addition, we noted that the contribution associated with changes in first birth transitions versus changes in unions to the declining first birth rate is, in general, similar across all SES groups. However, some differences emerged. First, the decline in first birth transitions among married individuals explained a larger share of the total first birth decline among women with a higher level of education. That is, the share explained is almost four times higher among those with a tertiary-level education (27%) than among those who completed primary education alone (7%). This reflects both the fact that the first birth transition probabilities for married individuals with primary-level education declined less as well as the difference in the population composition: a much higher proportion of more highly educated individuals were married (Appendix Fig. 8). In turn, the declining transition to single motherhood appeared more important for women with the least education.

Turning to union formation, declining cohabitation rates appear more important among women with the least education. The contribution of changes in cohabitation rates negatively (although slightly) associated with a tertiary level of education among women, meaning that cohabitation rates have, in fact, increased among this group. If such rates remained unchanged, first birth rates would have decreased slightly faster. This also results from the low number of more highly educated individuals at ages coinciding with decreases in the cohabitation rates. Here, again, the results for men are, in general, rather similar, yet some differences exist. For instance, the declining cohabitation rates among the least educated were slightly more important among men. In addition, the contribution of decreasing single parenthood did not concentrate within any educational group as they did among women.

### Summary of the Results

We observed a lower fertility in unions after 2010, a long-term decline in marriage rates and increasing dissolution rates among cohabiting couples. Lower fertility in unions explained around three-quarters of the total decline in first births, and the decreasing first birth transitions appeared more important among cohabiting couples than among married individuals. Consequently, changes in unions explained around one-quarter of the total decline in first births. Furthermore, lower marriage rates were more important than changes in cohabitation formation and cohabitation dissolution, and increases in cohabitation dissolution were more important than declining figures in cohabitation formation in explaining the first birth decline. Results were similar for both men and women.

Our findings were largely consistent across SES groups, whereby first births declined and childbearing within unions explained most of this decline in all groups. In agreement with the study by Comolli et al. (2020), the total decline in first births was stronger among less educated groups. Nevertheless, the decrease in the transition from cohabitation to marriage explained more of the decline among the most highly educated, while the decrease in the transition from single to cohabitation explained more of the decline among the least educated. We also compared the findings among income quartile groups as a robustness check (Appendix Figs. 9, 11): results remained relatively consistent regardless of whether education or income was used as an indicator of socioeconomic position. We note, however, that some differences between income groups were not as strong as those that emerged between educational groups. Moreover, while the probability of experiencing a first birth among cohabiting couples declined rather consistently across SES groups regardless of the measure, among married couples the decline was weaker among the least educated, potentially reflecting the selection of this small group.

## Discussion

This study investigated how the fertility decline in the 2010s in Finland associated with changes in fertility in unions and with changes in union formation and dissolution. Fertility mainly occurs in unions (Jalovaara & Fasang, 2017; Kiernan, 1999), and pinning down whether the fertility decline occurs within unions or because lack of unions is to zoom on the potential causes of the fertility decline. Using full-coverage Finnish register data, an incidence-based multistate model, and a counterfactual approach, we analysed the changes in the transition probabilities between relationship states among single, cohabitating, and married individuals, and in the transition to first birth among each relationship state in Finland from 2000 through 2018, and estimated the impact of these changes on the first birth decline in Finland during the 2010s. While patterns in unions explained some of the decline, the decreased transition to the first birth within unions mattered more.

First births in contemporary Finland have increasingly been born to cohabiting couples, whereby marriage has increasingly followed childbearing. In this current trend, however, the declining marriage rates since 2010 are not followed by increasing nonmarital births, as the decreases in first births among cohabiting couples represent the primary driver of declines in total first births. Instead, the tendency to remain cohabiting without transitioning to either marriage or the first birth has increased rapidly, accompanied by a slight increase in the risk of separation among younger cohabiting couples. A slower decline in the first birth transition among married as compared to cohabiting couples was expected, since marriage reflects a stronger commitment and promotes childbearing more strongly than cohabitation (Jalovaara & Miettinen, 2013; Miettinen & Rotkirch, 2008). It may be that childbearing intentions have declined among cohabiting and married couples in Finland. Finnish surveys indicate that the proportion voluntary childless has risen in Finland: the probability of having childfree ideals at age 25 was around 5% for the 1975–79 female cohort and around 20% for the 1990–1994 female cohort (Savelieva et al., 2021a, 2021b).

It also seems plausible that weaker childbearing intentions may have contributed to declining rates of union formation: hence, it might be that the lack of a first birth itself contributed to the decline in marriage. Marriage is strongly related to the family formation process so that couples who (wish to) have children often marry, and vice versa, marriage promotes the first birth (Brien et al., 1999; Steele et al., 2005). Consequently, the first birth decline attributed to declining marriage rates does not necessarily reflect a tendency to eventually marry less (unless the couples remain childless). Given that in the Nordic countries marriage increasingly occurs after the first birth rather than before (Jónsson, 2020), to better understand the causality of declining marriage and declining first births, future studies could investigate marriage patterns among parents as well.

The changes in family formation witnessed since the 1960s in the Nordic and other countries, such as postponing and declining fertility and the rise in alternative living arrangements compared to marriage, have often been explained by the second demographic transition theory (SDT), where the weakening of traditional family values gives rise to individual autonomy and self-actualisation (Lesthaeghe, 2014). Contrary to the predictions of SDT, the recent new family demographic developments in Finland were not necessarily driven by more highly educated individuals. Perhaps in Finland—a country with a long history of individualistic values (Sobotka, 2008)—these values have already spread from the higher social strata to the whole population by the 2010s. Interestingly, a characteristic SDT feature, voluntary childlessness, seems to have gained ground in Finland only in the last decade (Savelieva et al., 2021b) and may explain some of the fertility decline.

Increasing uncertainty that goes beyond the actual own economic circumstances has been suggested to drive the recent declining fertility trends in the Nordic (Comolli et al., 2020) and other European countries (Vignoli et al., 2020a, 2020b). The patterns we observed in Finland agree to some extent with this view. First, the declining fertility rates in unions explained the lion’s share of the decline in first births. This could be viewed as in line with the reasoning on uncertainty, given that uncertainty is less likely to be an obstacle to forming a cohabiting union than it is for more permanent and irreversible life decision like childbearing or marriage (Guetto et al., 2020). Also Finnish surveys suggest that perceived uncertainty is considered the most important subjective reason to postpone childbearing in the 2010s (Savelieva et al., 2021a). Second, the patterns remained largely consistent across SES groups, which would be generally not discrepant with the view of uncertainty. The sharper decline among the lower SES groups, as found in this study, however, suggests that actual economic constraints may not be completely irrelevant for the recent changes.

Although the decrease in the transition to cohabitation did not strongly explain the total decline in first births, its role should not be dismissed. First births remained rather unaffected by decreased cohabitation rates at younger ages, much due to the fact that first birth rates among young cohabitating couples are low. Moreover, a declining trend towards entering a cohabiting union remained notable primarily among the lower SES groups. Cohabitation rates have been stable in Finland for quite some time, but began declining at younger ages after 2015, a departure from the previous long-term trend. It is still unclear whether this merely reflects postponement or if we are also observing an increase in the share of individuals who never partner in the near future. The sharper decline in cohabitation rates among the lower SES groups, particularly among men, may imply that these groups are experiencing greater difficulties in the mating market. We agree with previous views arguing that the increase in the availability of dating partners through online dating sites and its effect on (un)stable union formation is an avenue for further investigation (Hiilamo, 2020).

If future transition probabilities remain at current levels and no catching up occurs, an inevitable consequence of the current first birth decline will result in increasing levels of ultimate childlessness. The expected proportion of childless individuals in Finland based on the first birth rates in 2010 and 2018, respectively, increased from 21.0% to 31.5% among women and from 28.7% to 41.5% among men. Given the sharp decline in childbearing within unions, future ultimate childlessness could potentially be related less to the absence of unions. Previously, ultimate childlessness in Finland has been linked to never partnering and to short or unstable spells of cohabitation (Jalovaara & Fasang, 2017), and rates of ultimate childlessness are low among married couples (Saarela & Skirbekk, 2019). Our findings suggest that all educational groups may witness increases in the rates of ultimate childlessness due to a declining progression to first births within unions alongside greater union instability, and the lower SES groups additionally due to the increasing difficulties in forming unions.

The primary strength of this study lies in our use of full-population Finnish register data, which distinguished nonmarital cohabitation from marriage. This distinction is crucial for understanding family-specific demographic changes within a context where cohabitation typically represents the first step in the family formation process and where first births are increasingly concentrated within cohabitating unions. A further strength lies in our analyses of data for both men and women, since analyses on men’s fertility remain rare. We also acknowledge several limitations in studying family formation based on educational attainment in the current period perspective, where the share enrolled (and in the decomposition analysis also the age structure) varies between groups with varying levels of attainment. Our sensitivity analysis (Appendix Fig. 12) revealed that the primary results regarding changes over time were quite similar regardless of whether we included currently enrolled or not. Reassuringly, the analysis by income group also indicated largely similar socioeconomic patterns in recent declines as those found from our analysis based on education.

Our model utilises annual transitions, given that it represents a straightforward way of proceeding and because personal information is available at the end of each calendar year. Potentially, using shorter transition periods for cohabitation, such as every three months, might prove appropriate, since that is the minimum time within which to capture unions in the registers. But, such short transitions may not be more informative vis-à-vis first birth transitions. An important question arises regarding whether we missed short-term changes in our approach. For instance, it is possible to observe a decline in the annual transition to cohabitation simply if the formation of cohabiting unions lasting shorter than one year increases while the total number of new cohabitations remains constant. Additional analyses revealed that the formation of unions lasting shorter than one year has also decreased in recent years (Appendix Table 2). In addition, our model did not take into account the duration of unions (Hoem et al., 2013; Jalovaara & Kulu, 2018). That said, additional analyses revealed that first birth rates have decreased, and separation rates have increased rather similarly regardless of union length (Appendix Fig. 13).

In conclusion, this study demonstrated that the sharp decline in first birth rates in the 2010s in Finland is associated with changes in partnering. However, the declining tendency to experience a first birth within unions is most important in explaining the fertility decline. Future studies should specifically focus on the declining tendency to transition to parenthood among cohabiting couples, as well as to the increasing instability of such unions.

### Electronic supplementary material

Below is the link to the electronic supplementary material.

Supplementary file1 (DOCX 17 kb)

## Figures and Tables

**Fig. 7:**
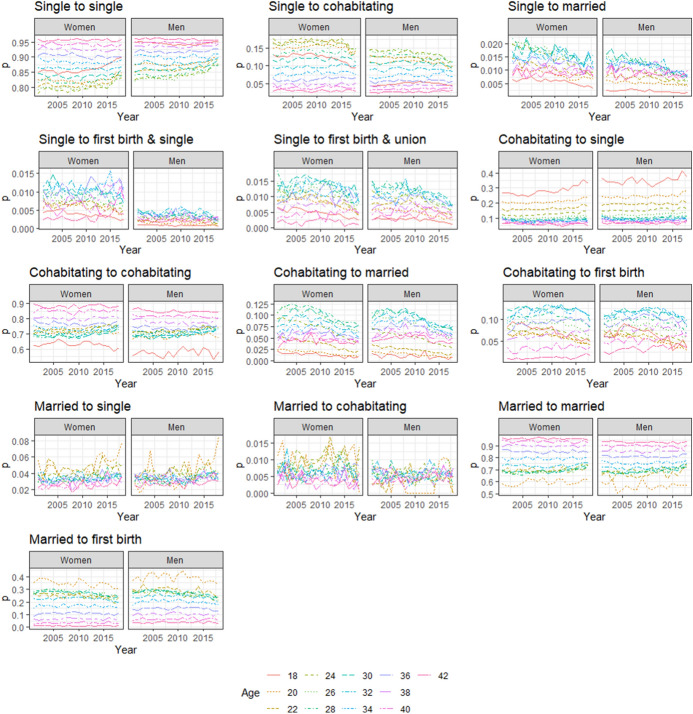
Age-specific transition probabilities for single, cohabitating, and married individuals, and the first birth among childless women and men in Finland, 2001–2018

**Fig. 8:**
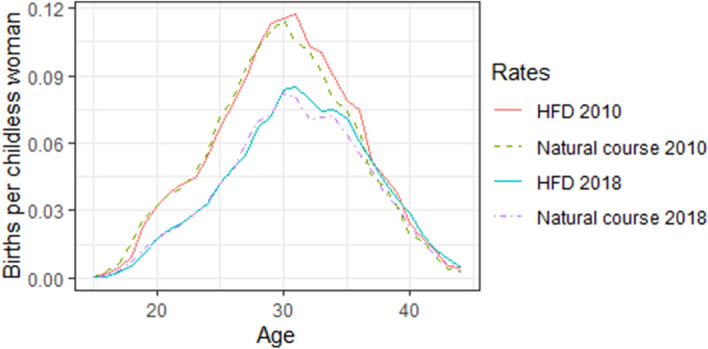
First birth rates (births per childless woman) based on the Human Fertility Database (HFD) and the natural course in 2010 and 2018

**Fig. 9:**
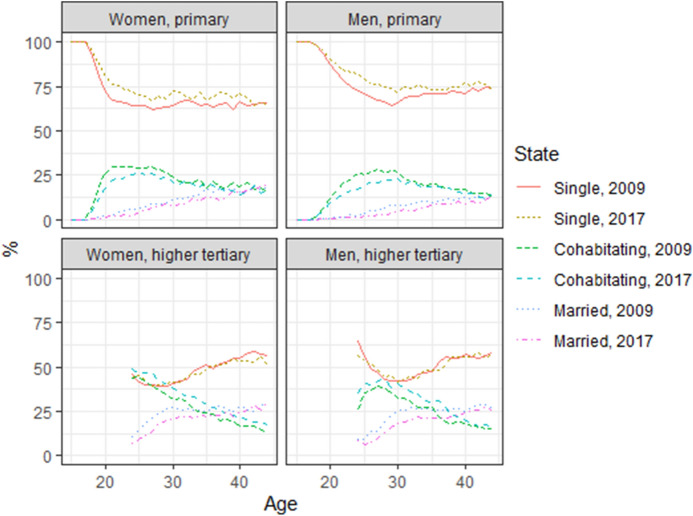
The population by partnership state in 2009 and 2017, for men and women with primary and higher tertiary education

**Fig. 10:**
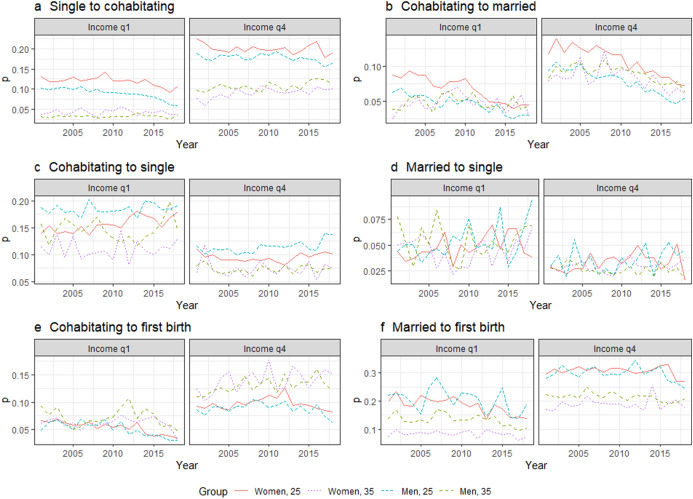
Transition probabilities for single, cohabitating, and married individuals, and first births among childless women and men in 2001–2018 by income quartile group at ages 25 and 35

**Fig. 11:**
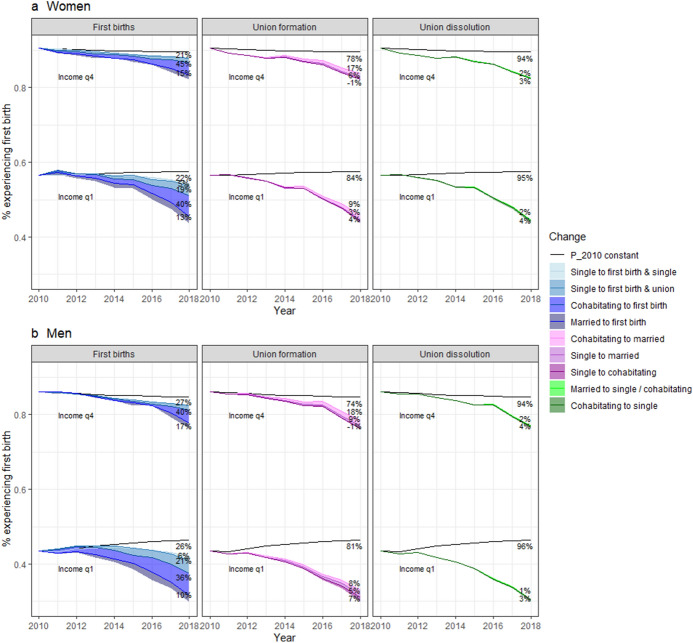
Contributions of declining first births, changes in union formations, and changes in union dissolutions to the decline in the percentage experiencing first births based on the first birth rates in 2010–2018 by income quartile groups. The top curve shows the results for the highest income group, while the bottom curve shows the results for the lowest income group

**Fig. 12:**
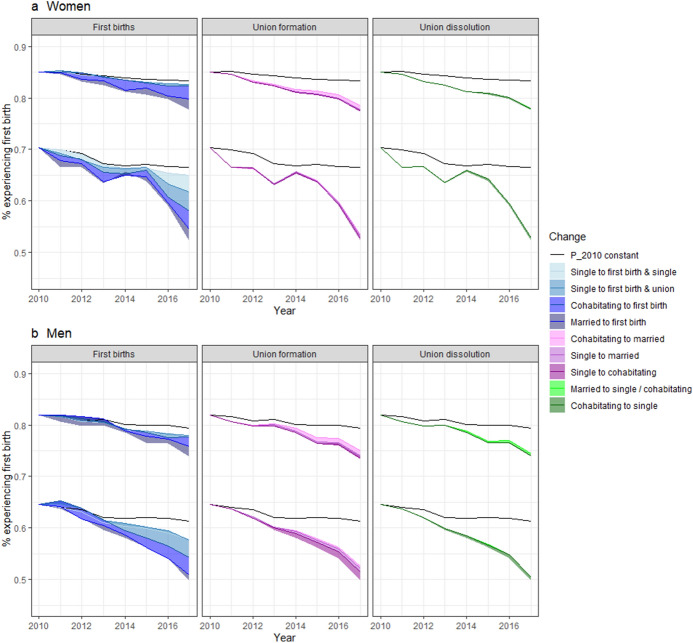
Contributions of declining first births, changes in union formations, and changes in union dissolutions to the decline in the percentage experiencing first births based on the first birth rates in 2010–2017 by education groups, excluding students. (Information regarding education enrolment was incomplete in 2018). The top curve shows the results for the most educated, whilst the bottom curve shows the results for the least educated

**Fig. 13:**
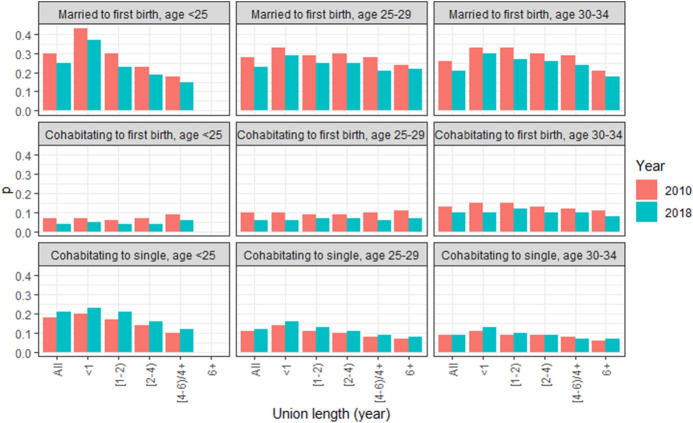
Transition probabilities by length of union for women aged < 25, 25–29 and 30–34, in 2010 and 2018. Length of union measured at the end of the previous year

## Data Availability

Individual level register data from Statistics Finland are not freely available.

## Appendix

See Figs. ^7^, 8, ^9^, 10, 11, 12, 13 and Tables 1 and 2.

**Table 1:** Number of first births, person-years of exposures, and partnership status transitions among the childless population, ages 15 to 44 among women and men, 2000–2018

	All	Women	Men
Characteristic	(*n* = 2 125 172)	(*n* = 993 024)	(*n* = 1 132 148)
First births, number	740 537	379 051	361 486
Person-years of follow-up	19 468 815	8 806 785	10 662 030
*Partnership transitions, number*			
Single to single	12 419 504	5 232 453	7 187 051
Single to cohabitating	1 206 555	604 671	601 884
Single to married	102 370	51 424	50 946
Single to first birth & single	47 249	31 398	15 851
Single to first birth & union	86 883	40 965	45 918
Cohabitating to single	536 569	268 414	268 155
Cohabitating to cohabitating	3 018 824	1 532 222	1 486 602
Cohabitating to married	269 197	136 150	133 047
Cohabitating to first birth	334 546	168 932	165 614
Married to single	47 898	24 330	23 568
Married to cohabitating	8 417	4 670	3 747
Married to married	1 118 944	573 400	545 544
Married to first birth	271 859	137 756	134 103

**Table 2:** Number of unions by starting year and union length, women aged < 25, 2000–2018

	>=1 year	[274–365) days	[183–274) days	[90–183) days	Total
2000	20,014	1191	1051	927	23,183
2001	20,755	1213	1095	838	23,901
2002	19,937	1215	1040	835	23,027
2003	19,827	1254	1035	857	22,973
2004	20,672	1143	1098	821	23,734
2005	20,543	1219	1071	838	23,671
2006	20,298	1247	1082	782	23,409
2007	20,001	1239	1041	765	23,046
2008	20,334	1273	1047	778	23,432
2009	20,526	1215	1108	745	23,594
2010	19,942	1267	1073	789	23,071
2011	20,243	1363	1092	801	23,499
2012	20,392	1296	1119	866	23,673
2013	20,115	1341	1095	804	23,355
2014	19,211	1260	1104	832	22,407
2015	19,042	1237	1069	829	22,177
2016	18,038	1326	1128	734	21,226
2017	15,886	1095	859	652	18,492
2018	15,969	1074	889	643	18,575

## Footnotes

1 Including individuals born abroad did not significantly change our primary findings.

2 Paternal information is delayed in the registers, meaning that there are more missing fathers to children born towards the end of the study period. Around 2% of all first births born in 2000–2010 had no registered father, while the proportion rose to around 6% for first births born in 2018.

3 When we refer to a specific year, we refer to the end of that specific year.

4 Fitting multinomial logistic regression models to the data would be an alternative, but the results would not differ.

5 Related methodological approaches have been applied only in few prior studies: hazard ratios were implemented in microsimulation models to link fertility to marital behaviours in Canada (Bélange et al., 2010), and a counterfactual approach was employed to examine the impact of union dissolution on fertility in Uruguay (Fernández Soto & Laplante, 2020).

6 Our counterfactual design does not necessarily take into account (changes in) the order of events. For instance, if we were to observe a first birth decline attributed to changes in declining marriage rates, it could in part reflect a tendency to marry increasingly after the first birth.

## Code Availability

The code is available upon request.

## References

[CR1] Ahn N, Mira P (2002). A note on the changing relationship between fertility and female employment rates in developed countries. Journal of Population Economics.

[CR2] Anderson T, Kohler H-P (2015). Low fertility, socioeconomic development, and gender equity. Population and Development Review.

[CR3] Andersson G, Rønsen M, Knudsen LB, Lappegård T, Neyer G, Skrede K, Teschner K, Vikat A (2009). Cohort fertility patterns in the Nordic countries. Demographic Research.

[CR4] Bastianelli, E., and Vignoli, D. (2021). The gendered relationship between (old and new forms of) employment instability and union dissolution, *Population Research and Policy Review*. 10.1007/s11113-021-09678-z.

[CR200] Becker, G. (1993). *A treatise on the family*. Harvard University Press.

[CR5] Bélanger, A., Jean-Dominique, M., and Spielauer, M. (2010). A microsimulation model to study the interaction between fertility and union formation and dissolution: An application to Canada and Quebec, *Canadian Studies in Population*, 37.

[CR6] Blossfeld, H.-P., and Hofmeister, H. (2006). *Globalization, uncertainty and women's careers: An international comparison*. Edward Elgar.

[CR7] Brewster K, Rindfuss RR (2000). Fertility and women's employment in industrialized nations. Annual Review of Sociology.

[CR8] Brien MJ, Lillard LA, Waite LJ (1999). Interrelated family-building behaviors: Cohabitation marriage, and nonmarital conception. Demography.

[CR9] Briggs A, Sculpher M (1998). An introduction to Markov modelling for economic evaluation. PharmacoEconomics.

[CR10] Comolli, C. L., Neyer, G., Andersson, G., Dommermuth, L., Fallesen, P., Jalovaara, M., Jónsson, A., Kolk, M., and Lappegård, T. (2020). Beyond the economic gaze: Childbearing during and after recessions in the Nordic countries. *European Journal of Population*, 37, 473–520. 10.1007/s10680-020-09570-0. 10.1007/s10680-020-09570-0 PMC7676408 33230356

[CR11] Corselli-Nordblad, L. , and Gereoffy, A. (2015). Archive: Marriage and birth statistics - new ways of living together in the EU. In *Eurostat*.

[CR12] Esping-Andersen G (2009). Incomplete revolution: Adapting welfare states to women's new roles.

[CR13] Esping-Andersen G, Billari FC (2015). Re-theorizing family demographics. Population and Development Review.

[CR14] Eurostat (2021). Divorce indicators, Accessed 7.1.2021. https://appsso.eurostat.ec.europa.eu/nui/show.do?dataset=demo_ndivind&lang=en.

[CR15] Fernández Soto M, Laplante B (2020). The effect of union dissolution on the fertility of women in Montevideo, Uruguay. Demographic Research.

[CR16] Frejka T (2008). Overview chapter 2: Parity distribution and completed family size in Europe: Incipient decline of the two-child family model?. Demographic Research.

[CR17] Frejka T, Goldscheider F, Lappegård T (2018). The two-part gender revolution, women’s second shift and changing cohort fertility. Comparative Population Studies.

[CR18] Goldin C (2006). The quiet revolution that transformed women's employment, education, and family. American Economic Review.

[CR19] Goldscheider F, Bernhardt E, Lappegård T (2015). The gender revolution: A framework for understanding changing family and demographic behavior. Population and Development Review.

[CR20] Guetto, R., Vignoli, D., and Bazzani, G. (2020). Marriage and cohabitation under uncertainty: The role of narratives of the future during the COVID-19 pandemic. *European Societies* 1–15.

[CR21] Hellstrand, J., Nisén, J., Miranda, V., Fallesen, P., Dommermuth, L., & Myrskylä, M. (2021). Not just later, but fewer: Novel trends in cohort fertility in the Nordic countries. *Demography*, *58*(4), 1373–1399. 10.1215/00703370-9373618 10.1215/00703370-9373618 34251453

[CR22] Hellstrand J, Nisén J, Myrskylä M (2020). All-time low period fertility in Finland: Demographic drivers, tempo effects, and cohort implications. Population Studies.

[CR23] Hiilamo H (2020). Why fertility has been declining in Finland after the global recession? : A theoretical approach. Finnish Yearbook of Population Research.

[CR24] Hoem JM, Jalovaara M, Muresan C (2013). Recent fertility patterns of Finnish women by union status: A descriptive account. Demographic Research.

[CR25] Human Fertility Database. (2019). www.humanfertility.org.

[CR26] Jalovaara M (2012). Socio-economic resources and first-union formation in Finland, cohorts born 1969–81. Population Studies.

[CR27] Jalovaara M (2013). Socioeconomic resources and the dissolution of cohabitations and marriages. European Journal of Population.

[CR28] Jalovaara M, Andersson G (2018). Disparities in children’s family experiences by mother’s socioeconomic status: The case of Finland. Population Research and Policy Review.

[CR29] Jalovaara M, Fasang AE (2017). From never partnered to serial cohabitors: Union trajectories to childlessness. Demographic Research.

[CR30] Jalovaara M, Fasang AE (2020). Family life courses, gender, and mid-life earnings. European Sociological Review.

[CR31] Jalovaara M, Kulu H (2018). Separation risk over union duration: An immediate itch?. European Sociological Review.

[CR32] Jalovaara M, Miettinen A (2013). Does his paycheck also matter?: The socioeconomic resources of co-residential partners and entry into parenthood in Finland. Demographic Research.

[CR33] Jalovaara M, Neyer G, Andersson G, Dahlberg J, Dommermuth L, Fallesen P, Lappegård T (2019). Education, gender, and cohort fertility in the Nordic countries. European Journal of Population.

[CR34] Jónsson, A. K. (2020). A nation of bastards? Registered cohabitation, childbearing, and first-marriage formation in Iceland, 1994–2013, *European Journal of Population*. 10.1007/s10680-020-09560-2. 10.1007/s10680-020-09560-2 PMC7865053 33597836

[CR35] Kemeny JG, Snell JL (1971). Finite Markov chains.

[CR36] Kennedy S, Bumpass LL (2008). Cohabitation and children's living arrangements: New estimates from the United States. Demographic Research.

[CR37] Kiernan K (1999). Childbearing outside marriage in Western Europe. Population Trends.

[CR38] Kolk M (2019). Weak support for a U-shaped pattern between societal gender equality and fertility when comparing societies across time. Demographic Research.

[CR39] Kravdal Ø (1994). The importance of economic activity, economic potential and economic resources for the timing of first Births in Norway. Population Studies.

[CR40] Kreyenfeld, M., and Konietzka, D. (2017). *Childlessness in Europe: Contexts, causes, and consequences*. Springer.

[CR41] Lesthaeghe R (2010). The unfolding story of the second demographic transition. Population and Development Review.

[CR42] Lesthaeghe R (2014). The second demographic transition: A concise overview of its development. Proceedings of the National Academy of Sciences of the United States of America.

[CR43] Lesthaeghe R, Surkyn J (1988). Cultural dynamics and economic theories of fertility change. Population and Development Review.

[CR44] Liefbroer AC (2005). The impact of perceived costs and rewards of childbearing on entry into parenthood: Evidence from a panel study. European Journal of Population.

[CR45] McDonald P (2000). Gender equity in theories of fertility transition. Population and Development Review.

[CR46] Miettinen, A., and Rotkirch, A. (2008). *Milloin on lapsen aika? Lastenhankinnan toiveet ja esteet [When is the right time for children. Expectations and barriers to childbearing]* (E 34. Helsinki: Family Federation of Finland, The Population Research Institute).

[CR47] Miettinen, A., Rotkirch, A., Szalma, I., Donno, A., and Tanturri, M.-L. (2015). *Increasing childlessness in Europe: Time trends and country differences*. Families And Societies project.

[CR48] Mills M, Blossfeld H-P (2003). Globalization, uncertainty and changes in early life courses. Zeitschrift Für Erziehungswissenschaft.

[CR49] Mills, M., and Blossfeld, H.-P. (2013). The second demographic transition meets globalization: A comprehensive theory to understand changes in family formation in an era of rising uncertainty. In *Ann Evans and Janeen Baxter* (eds.), *Negotiating the life course: Stability and change in life pathways*. Springer.

[CR50] Mills M, Rindfuss RR, McDonald P, te Velde E (2011). 'Why do people postpone parenthood? Reasons and social policy incentives. Human Reproduction Update.

[CR51] Myrskylä, M., Billari, F. C. and Kohler, H. P. (2011). High development and fertility: Fertility at older reproductive ages and gender equality explain the positive link, *Max Planck Institute for Demographic Research, Rostock, Germany. (MPIDR Working Paper WP-2011–017).*

[CR52] Myrskylä M, Kohler HP, Billari FC (2009). Advances in development reverse fertility declines. Nature.

[CR53] Mäenpää E, Jalovaara M (2015). 'Achievement replacing ascription? Changes in homogamy in education and social class origins in Finland. Advances in Life Course Research.

[CR54] Nisén J, Martikainen P, Myrskylä M, Silventoinen K (2018). Education, other socioeconomic characteristics across the life course, and fertility among finnish men. European Journal of Population.

[CR55] Nisén J, Martikainen P, Silventoinen K, Myrskylä M (2014). Age-specific fertility by educational level in the Finnish male cohort born 1940–1950. Demographic Research.

[CR56] OECD. (2021a). *Education at a glance 2021*.

[CR57] OECD. (2021b). OECD family database [electronic resource], Accessed 10.8.2021. http://www.oecd.org/social/family/database.htm.

[CR58] Official Statistics of Finland (OSF). (2018a). Births [e-publication]., Helsinki: Statistics Finland, Accessed 21.1.2019. https://www.stat.fi/til/synt/2017/synt_2017_2018-04-27_tie_001_en.html.

[CR59] Official Statistics of Finland (OSF). (2018b).Changes in marital status [e-publication], Helsinki: Statistics Finland, Accessed 7.5.2020. https://www.stat.fi/til/ssaaty/2018/ssaaty_2018_2019-06-18_kuv_001_en.html.

[CR60] Official Statistics of Finland (OSF). (2019). Decrease in birth rate slowed down in 2019, Helsinki: Statistics Finland, Accessed 31.8.2020. http://www.stat.fi/til/synt/2019/synt_2019_2020-04-24_tie_001_en.html.

[CR61] Official Statistics of Finland (OSF). (2021). Families [e-publication], Helsinki: Statistics Finland, Accessed 11.3.2021. http://www.stat.fi/til/perh/kas_en.html.

[CR62] Perelli-Harris B, Sigle-Rushton W, Kreyenfeld M, Lappegård T, Keizer R, Berghammer C (2010). The educational gradient of childbearing within cohabitation in Europe. Population and Development Review.

[CR63] Rotkirch, A. (2020). The wish for a child, *Vienna Yearbook of Population Research 2020*, 18. first online: 25.11.2020.

[CR64] Rotkirch, A., and Miettinen, A. (2017). Childlessness in Finland. In Michaela Kreyenfeld and Dirk Konietzka (Eds.), *Childlessness in Europe: Contexts, causes, and consequences*. Springer.

[CR65] Saarela, J., and Skirbekk, V. (2019). Childlessness and union histories: Evidence from Finnish population register data. *Journal of Biosocial Science*: 1–19. 10.1017/S0021932019000257 31169109

[CR66] Savelieva, K., Jokela, M., & Rotkirch, A. (2021a). Reasons to postpone or renounce childbearing during fertility decline in Finland. *SocArXiv*.

[CR67] Savelieva, K., Nitsche, N., Berg, V., Miettinen, A., Rotkirch, A., & Jokela, M. (2021b). Birth cohort changes in fertility ideals: Evidence from repeated cross-sectional surveys in Finland. *SocArXiv*.

[CR68] Sobotka T (2008). Overview chapter 6: The diverse faces of the second demographic transition in Europe. Demographic Research.

[CR69] Sobotka, T. (2017). Childlessness in Europe: Reconstructing long-term trends among women born in 1900–1972. In Michaela Kreyenfeld and Dirk Konietzka (Eds.), *Childlessness in Europe: Contexts, causes, and consequences*. Springer.

[CR70] Sobotka T, Skirbekk V, Philipov D (2011). Economic recession and fertility in the developed world. Population and Development Review.

[CR71] Steele F, Kallis C, Goldstein H, Joshi H (2005). The Relationship between childbearing and transitions from marriage and cohabitation in Britain. Demography.

[CR72] Surkyn J, Lesthaeghe R (2004). Value orientations and the second demographic transition (SDT) in Northern Western and Southern Europe: An update. Demographic Research.

[CR73] Thomson E, Eriksson H (2013). Register-based estimates of parents' coresidence in Sweden, 1969–2007. Demographic Research.

[CR74] Van Bavel J (2012). The reversal of gender inequality in education, union formation and fertility in Europe. Vienna Yearbook of Population Research.

[CR75] Van De Kaa DJ (1987). Europe's second demographic transition. Population Bulletin.

[CR76] Vignoli, D., Bazzani, G., Guetto, R., Minello, A., and Pirani, E. (2020a). Uncertainty and narratives of the future: A theoretical framework for contemporary fertility. In Robert Schoen (ed.), *Analyzing contemporary fertility*. Springer.

[CR77] Vignoli D, Guetto R, Bazzani G, Pirani E, Minello A (2020). A reflection on economic uncertainty and fertility in Europe: The narrative framework. Genus.

[CR78] Zeman K, Beaujouan É, Brzozowska Z, Sobotka T (2018). Cohort fertility decline in low fertility countries: Decomposition using parity progression ratios. Demographic Research.

