# Supplementary material for: Less Partnering, Less Children, or Both? Analysis of the Drivers of First Birth Decline in Finland Since 2010
Source: Eur J Popul. 2022 Feb 14;38(2):191–221. doi: 10.1007/s10680-022-09605-8 (PMC9127029; doi:10.1007/s10680-022-09605-8)
Supplement: Supplementary file 1 — Supplementary file1 (DOCX 17 kb) [file 10680_2022_9605_MOESM1_ESM.docx]

# Online Appendices (Electronic Supplementary Material)

## Technical appendix

The age-specific transition probabilities in a specific year from 2010 through 2018 were collected in the following $93 x 93$ dimension transition matrix:

$\tilde{\boldsymbol{M}}\boldsymbol{=}$ $\left( \begin{matrix} \begin{matrix} \boldsymbol{p}_{\boldsymbol{single\_single}} & \boldsymbol{p}_{\boldsymbol{cohabitation\_single}} & \boldsymbol{p}_{\boldsymbol{marriage\_single}} & \boldsymbol{0} & \boldsymbol{0} & \boldsymbol{0} \\ \boldsymbol{p}_{\boldsymbol{single\_cohabitation}} & \boldsymbol{p}_{\boldsymbol{cohabitation\_cohabitation}} & \boldsymbol{p}_{\boldsymbol{marriage\_cohabitation}} & \boldsymbol{0} & \boldsymbol{0} & \boldsymbol{0} \\ \boldsymbol{p}_{\boldsymbol{single\_marriage}} & \boldsymbol{p}_{\boldsymbol{cohabitation\_marriage}} & \boldsymbol{p}_{\boldsymbol{marriage\_marriage}} & \boldsymbol{0} & \boldsymbol{0} & \boldsymbol{0} \\ \boldsymbol{0} & \boldsymbol{p}_{\boldsymbol{cohabitation\_first\_birth}} & \boldsymbol{p}_{\boldsymbol{marriage\_first\_birth}} & \boldsymbol{1} & \boldsymbol{0} & \boldsymbol{0} \\ \boldsymbol{p}_{\boldsymbol{single\_single\_first\_birth}} & \boldsymbol{0} & \boldsymbol{0} & \boldsymbol{0} & \boldsymbol{1} & \boldsymbol{0} \\ \boldsymbol{p}_{\boldsymbol{single\_union\_first\_birth}} & \boldsymbol{0} & \boldsymbol{0} & \boldsymbol{0} & \boldsymbol{0} & \boldsymbol{1} \end{matrix} \end{matrix} \right)\boldsymbol{,}$

where $\boldsymbol{p}_{\boldsymbol{single\_single}}$, $\boldsymbol{p}_{\boldsymbol{single\_cohabitation}}$, $\boldsymbol{p}_{\boldsymbol{single\_marriage}}$, $\boldsymbol{p}_{\boldsymbol{cohabitation\_single}}$, $\boldsymbol{p}_{\boldsymbol{cohabitation\_cohabitation}}$, $\boldsymbol{p}_{\boldsymbol{cohabitation\_marriage}}$, $\boldsymbol{p}_{\boldsymbol{marriage\_single}}$, $\boldsymbol{p}_{\boldsymbol{marriage\_cohabitation}}\boldsymbol{,}$ and $\boldsymbol{p}_{\boldsymbol{marriage\_marriage}}$ are $30 x 30$ matrix blocks with non-zero elements only on the first subdiagonal. The $30 x 30$ matrix blocks contain the age-specific transition probabilities between single, cohabitating, and married individuals at ages 15 through 44. For instance, the element in column one and row two of $p_{ss}$ refers to the probability of remaining single between the ages of 15 and 16. Furthermore, $\boldsymbol{p}_{\boldsymbol{single\_single\_first\_birth}}$, $\boldsymbol{p}_{\boldsymbol{single\_union\_first\_birth}}$, $\boldsymbol{p}_{\boldsymbol{cohabitation\_first\_birth,}}$ and $\boldsymbol{p}_{\boldsymbol{marriage\_first\_birth}}$ are $1 x 30$ row vectors.

Finally, we decompose the decline in first births from 2010 through 2018 by transition probability using the first birth transition probabilities and the $90 x 90$ submatrix $\boldsymbol{M}$**,** which contains only the following transient states:

$\boldsymbol{M=}$ $\left( \begin{matrix} \begin{matrix} \boldsymbol{p}_{\boldsymbol{single\_single}} & \boldsymbol{p}_{\boldsymbol{cohabitation\_single}} & \boldsymbol{p}_{\boldsymbol{marriage\_single}} \\ \boldsymbol{p}_{\boldsymbol{single\_cohabitation}} & \boldsymbol{p}_{\boldsymbol{cohabitation\_cohabitation}} & \boldsymbol{p}_{\boldsymbol{marriage\_cohabitation}} \\ \boldsymbol{p}_{\boldsymbol{single\_marriage}} & \boldsymbol{p}_{\boldsymbol{cohabitation\_marriage}} & \boldsymbol{p}_{\boldsymbol{marriage\_marriage}} \end{matrix} \end{matrix} \right)\boldsymbol{.}$

This is achieved by arranging the population in 2010 as the $90 x 1$ matrix $\boldsymbol{N}$

$\boldsymbol{N=}\left( \begin{matrix} \begin{matrix} \boldsymbol{n}_{\boldsymbol{single}} \\ \boldsymbol{n}_{\boldsymbol{cohabitation}} \\ \boldsymbol{n}_{\boldsymbol{marriage}} \end{matrix} \end{matrix} \right)$**,**

where, $\boldsymbol{n}_{\boldsymbol{single}}$ is a $30 x 1$ matrix with the number of single individuals aged 15 to 44, $\boldsymbol{n}_{\boldsymbol{cohabitation}}$ is a $30 x 1$ matrix with the number of cohabiting individuals aged 15 to 44, and $\boldsymbol{n}_{\boldsymbol{marriage}}$ is a $30 x 1$ matrix with the number of married individuals aged 15 to 44. In the constant probability births scenario, we transformed the population in 2010 using ${\boldsymbol{N}_{\boldsymbol{y+1}}\boldsymbol{=}\boldsymbol{M}_{\boldsymbol{2010}}\boldsymbol{N}}_{\boldsymbol{y}}$**,** where $y\in(2011,\ldots, 2018)$. The age-specific first birth rates are calculated as

$\boldsymbol{ASFB=(}{\boldsymbol{n}_{\boldsymbol{single}}\boldsymbol{p}_{\boldsymbol{single\_single\_first\_birth}}\boldsymbol{+}\boldsymbol{n}_{\boldsymbol{single}}\boldsymbol{p}_{\boldsymbol{single\_union\_first\_birth}}\boldsymbol{+ n}}_{\boldsymbol{cohabitation}}\boldsymbol{p}_{\boldsymbol{cohabitation\_first\_birth}}\boldsymbol{+}\boldsymbol{n}_{\boldsymbol{marriage}}\boldsymbol{p}_{\boldsymbol{marriage\_first\_birth}}\boldsymbol{)/(}\boldsymbol{n}_{\boldsymbol{single}}\boldsymbol{+}\boldsymbol{n}_{\boldsymbol{cohabitation}}\boldsymbol{+}\boldsymbol{n}_{\boldsymbol{marriage}}\boldsymbol{)}$**.**

At each step, the population ages. Thus, when individuals are aged 15 to 44 in year t, they will be aged 16 to 44 in year t + 1 (45-year-olds are automatically dropped from the sample). Therefore, in year t + 1, we add to the population the number of 15-year-old individuals, all of whom are considered single. Based on the age-specific first birth rates in each year, we calculate the annual proportion ever having a first birth for a synthetic cohort of women following a life-table approach. In the natural course births scenario, we proceed similarly, but transform the population using ${\boldsymbol{N}_{\boldsymbol{y+1}}\boldsymbol{=}\boldsymbol{P}_{\boldsymbol{y}}\boldsymbol{N}}_{\boldsymbol{y}}$.

We decompose the difference between constant probability births and natural course births step-by-step by keeping one of the transition probabilities at the 2010 level, while allowing the remainder of the transition probabilities to change according to the natural course. In order for the probabilities from an initial state to sum up to 1, we adjust the transition probability remaining in that initial state as follows:

For the first birth transitions:

1. $\boldsymbol{p}_{\boldsymbol{marriage\_marriage,y}}\boldsymbol{=1-}\boldsymbol{p}_{\boldsymbol{marriage\_first\_birth,2010}}\boldsymbol{-}\boldsymbol{p}_{\boldsymbol{marriage\_single,y}}\boldsymbol{-}\boldsymbol{p}_{\boldsymbol{marriage\_cohabitation,y}}$**;**
2. $\boldsymbol{p}_{\boldsymbol{cohabitation\_cohabitation,y}}\boldsymbol{=1-}\boldsymbol{p}_{\boldsymbol{cohabitation\_first\_birth,2010}}\boldsymbol{-}\boldsymbol{p}_{\boldsymbol{cohabitation\_single,y}}\boldsymbol{-}\boldsymbol{p}_{\boldsymbol{cohabitation\_marriage,y}}$**;**
3. $\boldsymbol{p}_{\boldsymbol{single\_single,y}}\boldsymbol{=1-}\boldsymbol{p}_{\boldsymbol{single\_union\_first\_birth,2010}}\boldsymbol{-}\boldsymbol{p}_{\boldsymbol{single\_single\_first\_birth,y}}\boldsymbol{-}\boldsymbol{p}_{\boldsymbol{single\_cohabitation,y}}\boldsymbol{-}\boldsymbol{p}_{\boldsymbol{single\_marriage,y}}\boldsymbol{;}$
4. $\boldsymbol{p}_{\boldsymbol{single\_single,y}}\boldsymbol{=1-}\boldsymbol{p}_{\boldsymbol{single\_union\_first\_birth,2010}}\boldsymbol{-}\boldsymbol{p}_{\boldsymbol{single\_single\_first\_birth,2010}}\boldsymbol{-}\boldsymbol{p}_{\boldsymbol{single\_cohabitation,y}}\boldsymbol{-}\boldsymbol{p}_{\boldsymbol{single\_marriage,y}}$**.**

For union formation:

1. $\boldsymbol{p}_{\boldsymbol{single\_single,y}}\boldsymbol{=1-}\boldsymbol{p}_{\boldsymbol{single\_cohabitation,2010}}\boldsymbol{-}\boldsymbol{p}_{\boldsymbol{single\_marriage,y}}\boldsymbol{-}\boldsymbol{p}_{\boldsymbol{single\_union\_first\_birth,y}}\boldsymbol{-}\boldsymbol{p}_{\boldsymbol{single\_single\_first\_birth,y}}$**;**
2. $\boldsymbol{p}_{\boldsymbol{single\_single,y}}\boldsymbol{=1-}\boldsymbol{p}_{\boldsymbol{single\_cohabitation,2010}}\boldsymbol{-}\boldsymbol{p}_{\boldsymbol{single\_marriage,2010}}\boldsymbol{-}\boldsymbol{p}_{\boldsymbol{single\_union\_first\_birth,y}}\boldsymbol{-}\boldsymbol{p}_{\boldsymbol{single\_single\_first\_birth,y}}$**;**
3. $\boldsymbol{p}_{\boldsymbol{cohabitation\_cohabitation,y}}\boldsymbol{=1-}\boldsymbol{p}_{\boldsymbol{cohabitation\_marriage,2010}}\boldsymbol{-}\boldsymbol{p}_{\boldsymbol{cohabitation\_single,y}}\boldsymbol{-}\boldsymbol{p}_{\boldsymbol{cohabitation\_first\_birth,y}}$**.**

For union dissolution:

1. $\boldsymbol{p}_{\boldsymbol{cohabitation\_cohabitation,y}}\boldsymbol{=1-}\boldsymbol{p}_{\boldsymbol{cohabitation\_single,2010}}\boldsymbol{-}\boldsymbol{p}_{\boldsymbol{cohabitation\_marriage,y}}\boldsymbol{-}\boldsymbol{p}_{\boldsymbol{cohabitation\_first\_birth,y}}$**;**
2. $\boldsymbol{p}_{\boldsymbol{marriage\_marriage,y}}\boldsymbol{=1-}\boldsymbol{p}_{\boldsymbol{marriage\_single,2009}}\boldsymbol{-}\boldsymbol{p}_{\boldsymbol{marriage\_cohabitation,2010}}\boldsymbol{-}\boldsymbol{p}_{\boldsymbol{marriage\_first\_birth,y}}$**.**

In the analysis of income groups, we proceeded similarly as we did for the total population. However, we considered 17 as the minimum age, since the majority of individuals had no or low incomes at younger ages. As the population ages, we add to the population the number of 17-year-old individuals in that income group.

For the educational groups, we proceeded as follows. We calculated the proportion moving to a higher education level in each year. When we transformed the population in the counterfactual approach, we removed the individuals moving to a higher education level based on the calculated rates. For those with a primary education, each year we add to the population the number of 15-year-old individuals; but, among the higher education groups, in year t + 1, we add the number of individuals with a lower education in year t at all ages and for all partnership statuses. For instance, those individuals with a primary education in year t but secondary education in t + 1 were added to the population of individuals with a secondary education in year t + 1. Similarly, in the sensitivity analysis excluding students, we removed the proportion beginning their studies, and added to the population those who graduated. Furthermore, we considered secondary education from age 18, lower tertiary education from age 22, and higher tertiary education from age 24 to avoid unstable rates.
